# Is 3D Printing Promising for Osteochondral Tissue Regeneration?

**DOI:** 10.1021/acsabm.3c00093

**Published:** 2023-03-21

**Authors:** Duygu Ege, Vasif Hasirci

**Affiliations:** † Institute of Biomedical Engineering, Boğaziçi University, Rasathane Cd, Kandilli Campus, Kandilli Mah., 34684 Istanbul, Turkey; ^‡^Biomaterials A & R Ctr, and ^§^Department of Biomedical Engineering, Acibadem Mehmet Ali Aydinlar University, Kayisdagi Ave., Atasehir, 34684 Istanbul, Turkey; ∥ Center of Excellence in Biomaterials and Tissue Engineering, METU Research Group, BIOMATEN, Cankaya, 06800 Ankara, Turkey

**Keywords:** 3D printing, ostechondral tissue, GelMA, alginate, cartilage

## Abstract

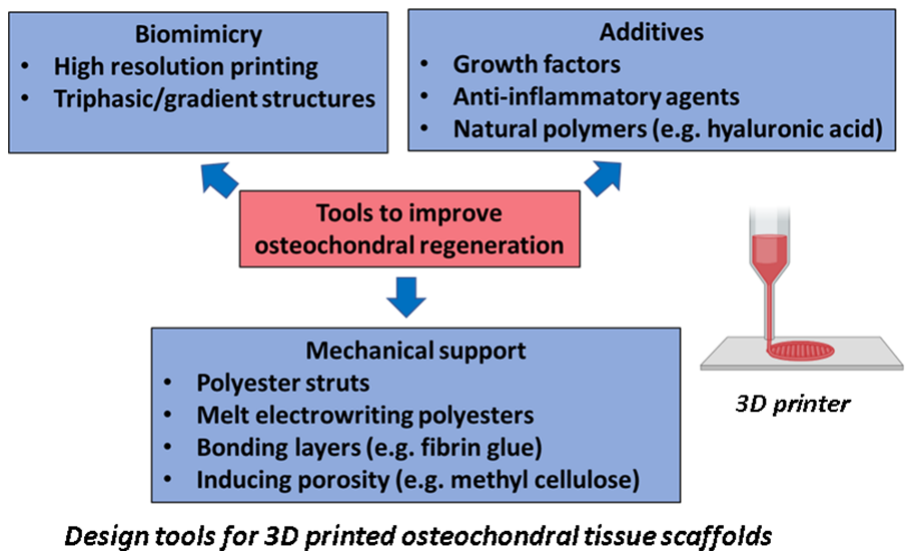

Osteochondral tissue regeneration is quite difficult to achieve due to the complexity of its organization. In the design of these complex multilayer structures, a fabrication method, 3D printing, started to be employed, especially by using extrusion, stereolithography and inkjet printing approaches. In this paper, the designs are discussed including biphasic, triphasic, and gradient structures which aim to mimic the cartilage and the calcified cartilage and the whole osteochondral tissue closely. In the first section of the review paper, 3D printing of hydrogels including gelatin methacryloyl (GelMa), alginate, and polyethylene glycol diacrylate (PEGDA) are discussed. However, their physical and biological properties need to be augmented, and this generally is achieved by blending the hydrogel with other, more durable, less hydrophilic, polymers. These scaffolds are very suitable to carry growth factors, such as TGF-β1, to further stimulate chondrogenesis. The bone layer is mimicked by printing calcium phosphates (CaPs) or bioactive glasses together with the hydrogels or as a component of another polymer layer. The current research findings indicate that polyester (i.e. polycaprolactone (PCL), polylactic acid (PLA) and poly(lactide-*co*-glycolide) (PLGA)) reinforced hydrogels may more successfully mimic the complex structure of osteochondral tissue. Moreover, more recent printing methods such as melt electrowriting (MEW), are being used to integrate polyester fibers to enhance the mechanical properties of hydrogels. Additionally, polyester scaffolds that are 3D printed without hydrogels are discussed after the hydrogel-based scaffolds. In this review paper, the relevant studies are analyzed and discussed, and future work is recommended with support of tables of designed scaffolds. The outcome of the survey of the field is that 3D printing has significant potential to contribute to osteochondral tissue repair.

## 1 Introduction

**Figure 1:**
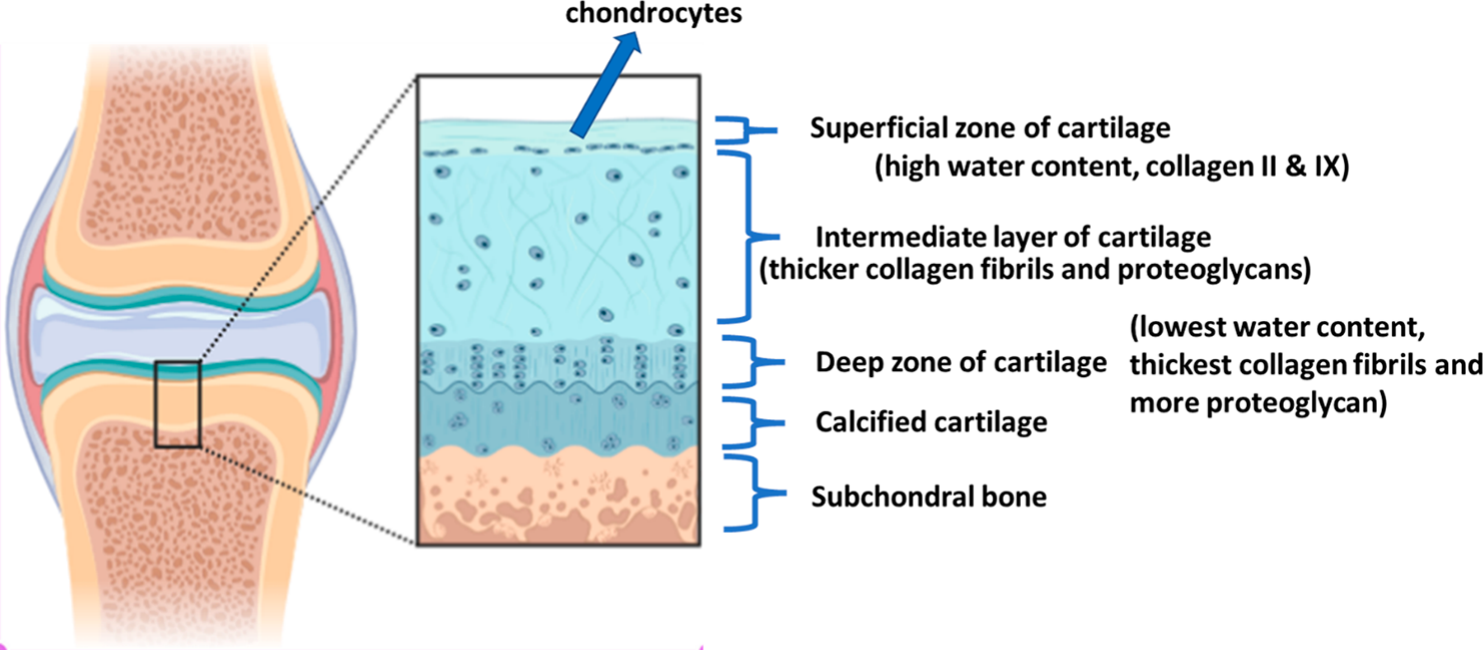
Complex structure of osteochondral tissue^5^ (drawn in with combination of Biorender software and Power Point Presentation).

Osteochondral tissue bears loads while maintaining motion without friction.^1−4^ Articular cartilage has a thickness of 2–4 mm with regions of superficial, intermediate, and deep zones. The superficial zone of cartilage has a high water content, and it has collagen II and IX fibrils. In this region, the chondrocytes are flattened. The intermediate zone has a relatively lower water content with proteoglycans and thicker collagen fibrils. More spherical chondrocytes are present in this layer. The deep zone has the lowest water content with the thickest collagen fibrils and more proteoglycan. This layer contains columnar chondrocytes.^5^ Between the cartilage and subchondral bone, there is calcified cartilage.^6^ Over this tissue, the physical, mechanical and biological properties vary significantly. Subchondral bone is highly vascularized tissue which is a complex interface between bone and cartilage.^7−9^ Due to easy access to nutrients, osteochondral bone tissue may regenerate more easily than cartilage.^10^ However, due to the avascular and aneural characteristics of cartilage and the complexity of the interface between bone and cartilage, regeneration of osteochondral tissue is challenging.^11^ For successful regeneration of this tissue, regeneration of cartilage and bone should take place at once.^12−15^Figure 1 shows the osteochondral tissue structure.

Osteochondral defects may occur due to inflammation or osteoarthritis, which lead to damage of the cartilage and underlying bone.^16−20^ Unfortunately, cartilage has limited capability to self-repair, and the disease causes degradation of articular (hyaline) cartilage and narrowing of joint space.^21,22^ Currently, the gold standard for treatment of osteochondral defects is surgery.^18^ Usually tissue is replaced with autografts, and bone tissue engineering implants are also used.^17,23^ Cells may also be injected to the defect site without a scaffold; however, this does not work very efficiently.^24^

3D printing comes forth as a promising technology to print anatomically matched implants.^25^ In this way, the rigidity, diffusivity, and tissue density of articular cartilage may be more precisely mimicked.^7,26−29^ Currently the three most popular 3D printing techniques of hydrogels are (i) extrusion-based (fused deposition modeling (FDM),^30^ low temperature deposition modeling (LDM)), (ii) vat-polymerization (stereolithography (SLA), digital light processing (DLP)),^31^ and (iii) jetting-based (electrohydrodynamic jet printer).^5,32−34^ Moreover, cells are encapsulated in 3D printed scaffolds to enable more effective treatment of osteochondral defects.^35^

**Figure 2:**
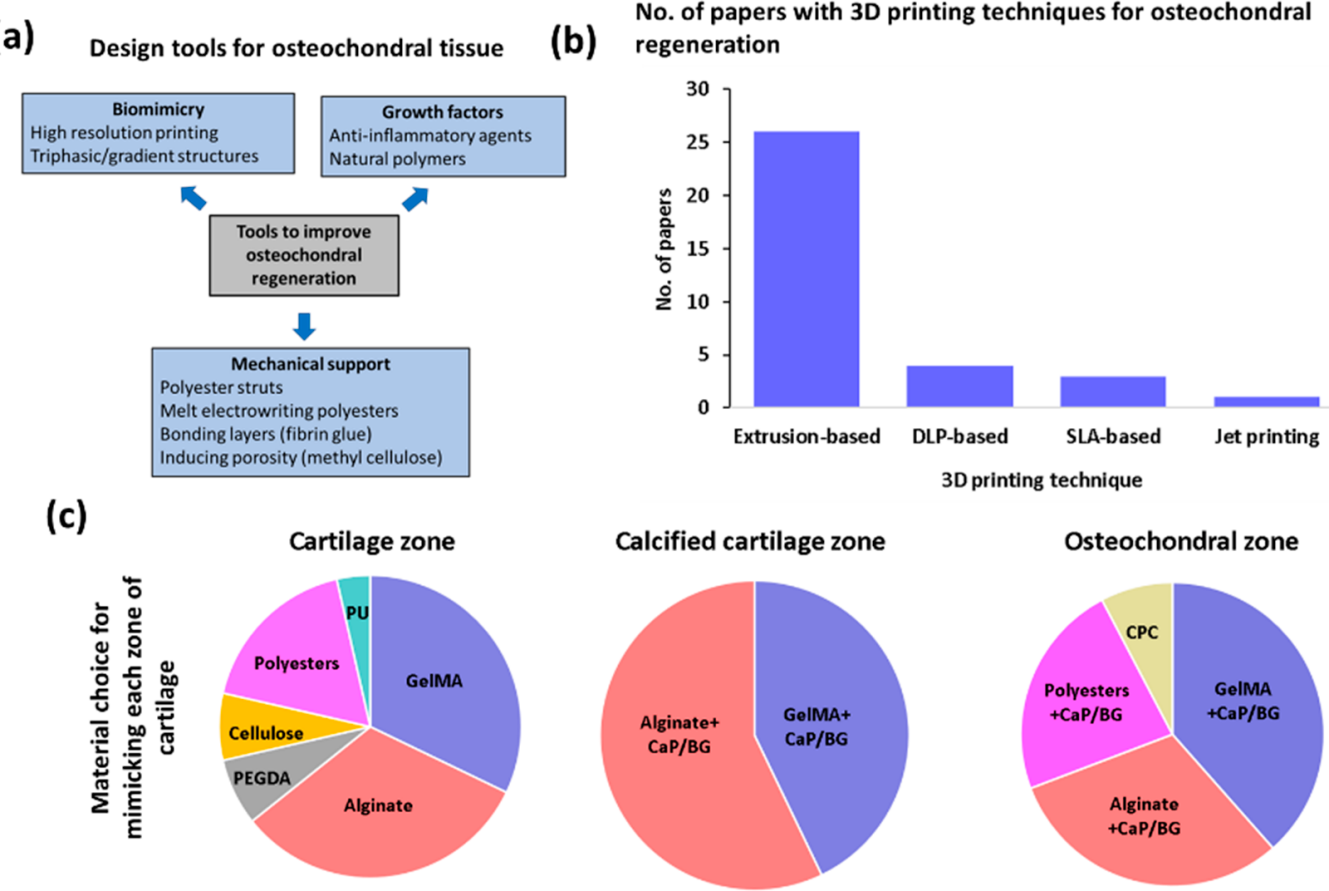
Strategies to produce osteochondral scaffolds: (a) tools for osteochondral regeneration; (b) no. of papers exploiting different 3D printing methods (drawn in PowerPoint); and (c) most frequently used biomaterials for cartilage, calcified cartilage, and subchondral bone layers in terms of their relevant percentages (drawn in PowerPoint).

In the studies on osteochondral research with 3D printing, the most exploited biomaterials were alginate, gelatin methacryloyl (GelMa), polyethylene glycol diacrylate (PEGDA)-based hydrogels, and polyester scaffolds (polycaprolactone (PCL), polylactic acid (PLA) and poly(lactide-*co*-glycolide) (PLGA)). These common biomaterials are reinforced with other materials to further reinforce their physical and biological properties.^36−38^ To 3D print scaffolds, some factors should be taken into consideration. The printing and gelation properties of the biomaterials should be optimized to achieve mechanically stable samples with high fidelity. Also, the entrapped cells should not undergo mechanical damage in the hydrogel.^39^ The printing pressure, temperature, viscosity of the bioink, and diameter of the nozzle should be optimized to improve printing resolution, mechanical properties, and cell viability.^40^Figure 2(a), (b), and (c) shows the summary of strategies for 3D printing of scaffolds for osteochondral repair, number of research papers for different 3D printing techniques, and material choice for each zone, respectively.

Figure 2(a) shows strategies for improving osteochondral regeneration. By biomimicry, addition of additives, and enhancing mechanical support, osteochondral regeneration may improve. Figure 2(b) shows that the most commonly used 3D printing method is extrusion-based (∼77% of the research papers), whereas DLP, SLA and jet printing methods are very rarely studied in the literature so far. As can be seen from Figure 2(c), natural hydrogels are commonly exploited for osteochondral regeneration instead of synthetic hydrogels (such as poly(vinyl alcohol) (PVA) and polyethylene glycol (PEG)) due to their cell instructive cues.^41−45^ For calcified and osteochondral zones, these polymers are often reinforced with either calcium phosphates or bioactive glasses. The w/w% values of calcium phosphates and bioactive glasses increase from the calcified cartilage zone to the osteochondral zone.^46^

**Figure 3:**
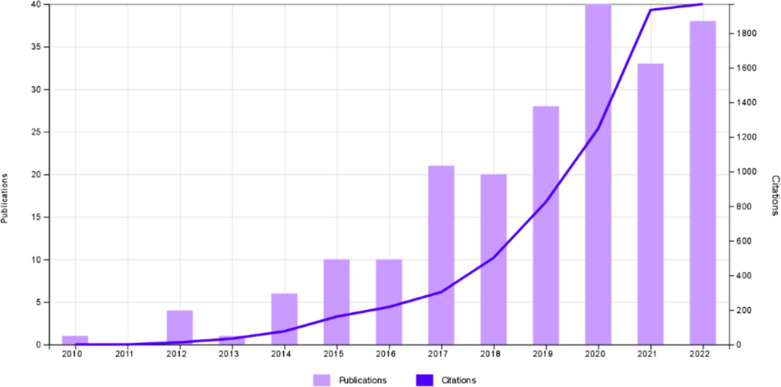
Number of research papers and citations on 3D printing of scaffolds for osteochondral regeneration (extracted from Web of Science in 2022).

For preparation of this review paper, a search from 2000 through 2023 was carried out with the search engines Google Scholar, Scopus and Web of Science with the keywords osteochondral tissue regeneration, 3D printing, and bioprinting. Figure 3 shows that the number of studies on 3D printing of scaffolds for osteochondral repair increased steeply only after 2010. In this review paper, the most commonly 3D printed constructs involving hydrogels for osteochondral repair are discussed in the sections on GelMA and alginate hydrogels. This is followed by a section on the constructs with other studied hydrogels. In the final section, research on 3D printing of polyesters without the use of hydrogels is presented. Finally, limitations and future perspectives on 3D printing of osteochondral scaffolds are delivered with the aid of tables and diagrams.

## 2 Hydrogels

In this section, the hydrogels produced for osteochondral regeneration are presented. In the first two subsections scaffolds fabricated with GelMA and alginate hydrogels, which are the most commonly studied hydrogels for osteochondral regeneration, will be discussed. Then, in the third subsection, other common hydrogels including PEGDA, cellulose-based hydrogels, polyurethane (PU)-based hydrogels, methylated hyaluronic acid (HAMA), chondroitin sulfate, fibrin-based hydrogels, and chitosan-based hydrogels will be explained.

### 2.1 GelMA Hydrogels

In many of the studies, cell laden bioinks are used as precursors for 3D printing hydrogels.^43^ GelMA was introduced in 2000, and it is one of the most commonly studied hydrogels.^47^ GelMA is a gelatin derivative and consists of methacryloyl (methacrylamide and methacrylate) side groups.^48^ It has high cell adhesion due to its arginine-glycine-aspartic acid (RGD) sequences.^49^ The immunogenicity of GelMA is lower than those of pure gelatin and collagen.^27^ GelMA hydrogels maintain controlled release of growth factors as well as promotion of cell attachment and differentiation toward chondrogenesis.^50,51^ Many studies indicate that human chondrocytes in GelMA-based scaffolds produce cartilage-specific components of glycosaminoglycans (GAGs) and Col2-α1. However, they are not mechanically durable.^52^

**Figure 4:**
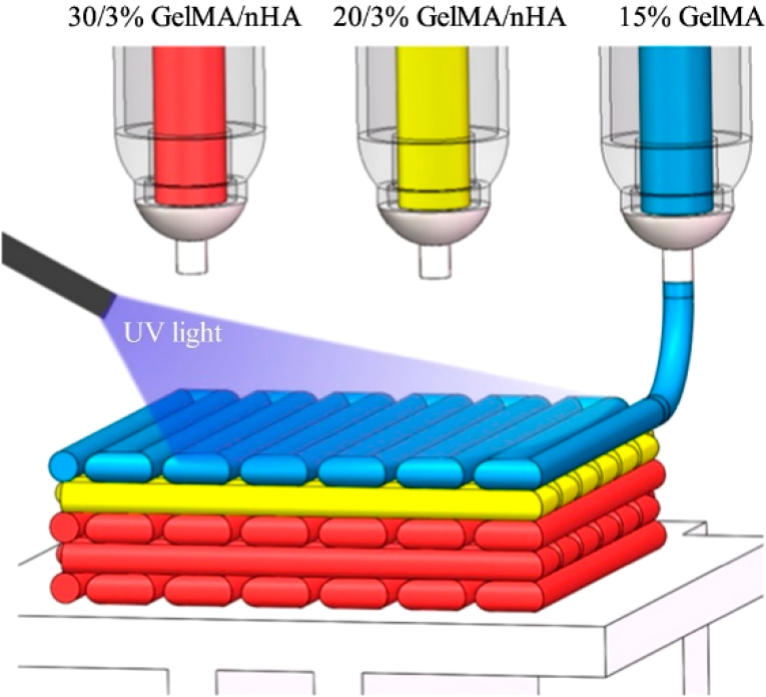
Schematic diagram showing the multi-nozzle extrusion-based 3D printing system for subchondral tissue and its cross-linking under UV light^2^ (Reproduced with permission from ref (2). Copyright 2019 Elsevier Publications).

Li et al.^2^ fabricated a triphasic scaffold with multi-nozzle extrusion-based 3D printing at 25 °C by 15% GelMA, 20 or 30% (w/w) GelMA (with 3% (w/w) nano-sized HAp) for cartilage, calcified cartilage, and osteochondral layers, respectively. GelMA precursor solution was prepared with 15, 20 and 30% (w/v) in phosphate buffer saline (PBS) solution with 0.05% (lithium phenyl-2,4,6-trimenthylbenzoyl phosphinate, C_16_H1_6_LiO_3_P) LAP photoinitiator. After 3D printing, the scaffolds were cross-linked by exposure to UV light (2 W/cm^2^) for 2 min. Physical characterization and *in vivo* studies with New Zealand white (NZW) rabbits indicated that these scaffolds were successful in repairing cartilage and subchondral bone simultaneously.^2^Figure 4 shows the 3D printing of this scaffold and its subsequent cross-linking with UV exposure.

Hyaluronic acid triggers chondrogenesis; however, it is not stable under *in vitro* conditions.^53^ To stabilize hyaluronic acid, it is entrapped in a GelMA hydrogel to form a semi-interpenetrating network.^1,54^ In the study of Chen et al.,^54^ 3D printed scaffolds were prepared with GelMA and 2% hyaluronic acid. Hydrogen bonding between hyaluronic acid and GelMA and cross-linking of GelMA increased the stability of the hydrogels and shape fidelity. A porous hyaluronic acid and hyaluronic acid with dynamically exchangeable hydrazone cross-linking were fabricated for bone and cartilage layers, respectively. The study indicated that the expression levels of chondrogenic marker genes (Col2-α1 and Sox-9 of rat bone marrow-derived stem cells (rBMSCs) cultured on dynamic hyaluronic acid hydrogel were increased significantly compared to other study groups. Moreover, the porous hyaluronic acid layer significantly promoted the expression of bone-related genes (Col1 and RUNX 2). It was explained that the dynamically exchangeable hydrazone cross-linked hyaluronic acid might have promoted the chondrogenic capacity of the dynamic hyaluronic acid component. This led to a high viscoelasticity similar to that of the cartilage matrix.^54^

**Figure 5:**
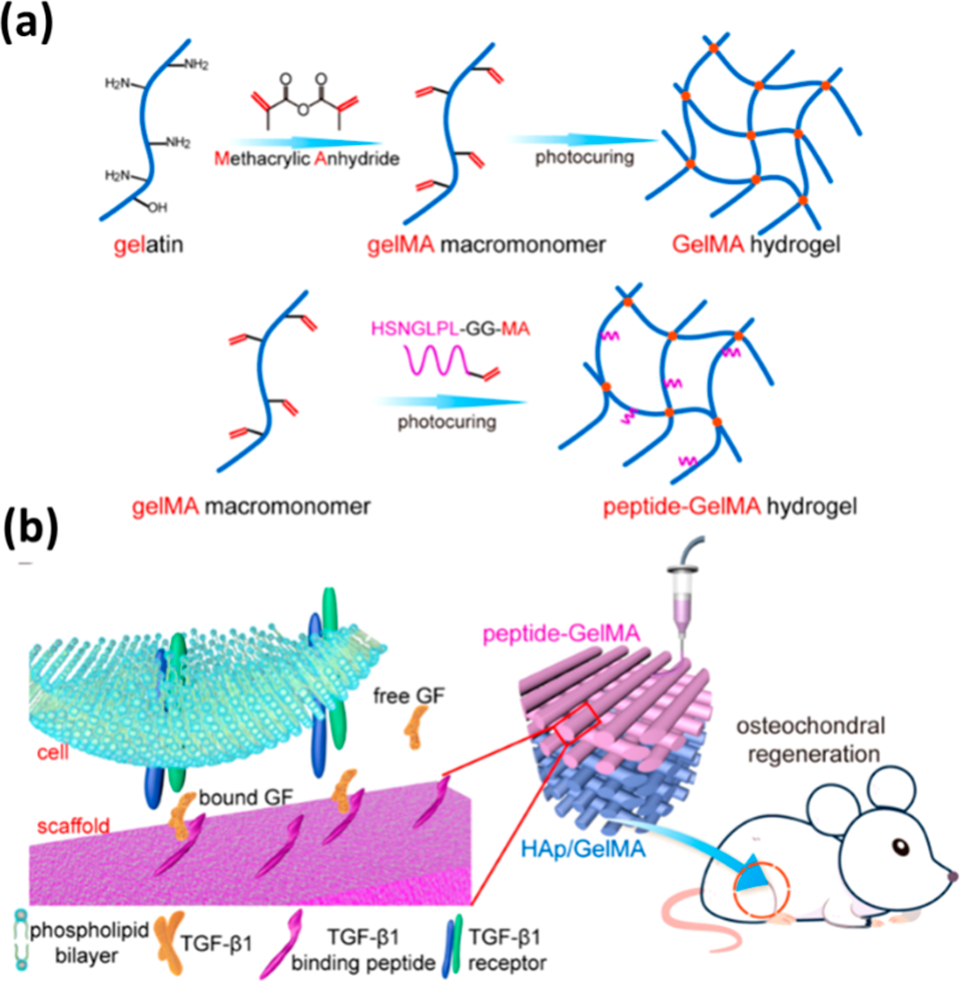
Bilayered GelMA scaffolds for osteochondral regeneration: (a) production of cross-linked GelMA hydrogel from GelMA macromonomer with TGF-β1 binding peptide (HSNGLPL-GG-MA); (b) scheme showing bound and free growth factors (GFs) with recruitment of TGF-β1 *in vivo* (left) and implantation of a bilayer scaffold of peptide-GelMA and HAp/GelMA in a rat model (right)^57^ (Reproduced with permission from ref (57). Copyright 2022 ACS Publications).

TGF-ß1 is a TGF-ß family member, and it is a potent stimulator of chondrogenesis, which can significantly induce Sox-9 expression and enhance cartilaginous extracellular matrix (ECM) production.^18,52,55^ TGF-ß1 binding peptide may recruit endogenous growth factors such as TGF-ß1.^56^ Therefore, in the study of Ding et al.,^57^ a different approach was taken to maintain a controlled amount of TGF-ß1 in the extrusion-based 3D printed (Regenova Ink) bilayered porous scaffold. 10% GelMA bioink was prepared in PBS buffer with photoinitiator I2959. 3D printing was conducted with a speed of 30 mm/s at 20 °C which was then UV cross-linked (365 nm) for 5 min. The hydrogel was bound to a peptide to stimulate chondrogenic regeneration. In the bone layer, 10% GelMA bioink was incorporated with 1% nano-sized HAp to stimulate osteogenesis. Figure 5 shows this design in detail.

*In vivo* study with Sprague–Dawley rats indicated that the mean subchondral peptide-bone scores in the hydrogel group were on average higher than those in the GelMA group. Another strategy to incorporate growth factors is to incorporate platelet-rich plasma (PRP) in the hydrogel. PRP has fibronectin, cytokines, and growth factors such as TGF-β, IGF1, β-FGF, and Platelet-derived growth factor AB (PDGF-AB) which stimulate chondrogenesis.^27^ PRP is also known to promote chondrogenesis.^58−60^ In the study of Jiang et al.,^27^ to maintain chemokines and growth factors at the injury site, the authors 3D printed 10, 20, and 50% PRP incorporated GelMA (15 wt %/v) hydrogels with a SLA-based 3D printer. 0.05% LAP was combined with GelMA solution before 3D printing. The hydrogel was cross-linked with visible light source 11 mW/cm^2^ for 30 s. The structure had an internal pore size of 127 ± 5 μm with a porosity of 75%. Incorporation of PRP was shown to induce chondrogenesis of BMSCs. The study indicated that PRP had more unknown growth factors which further stimulated chondrogenesis. The results indicated that PRP/GelMA hydrogels had a better histological outcome than pure GelMA hydrogels.

Extrusion-based printing is sufficient to mimic the subchondral bone in terms of its mechanical and biological properties. However, its resolution is limited with the size of the nozzle, the printing speed and the distance between the nozzle and printing layer.^61^ Therefore, extrusion printers are not very successful in mimicking the cartilage and calcified cartilage layers of osteochondral tissue.^62^ DLP has a resolution of around 50 μm, which is higher than the resolution of extrusion-based printers (150 μm). To produce more complex shapes which cannot be printed with an extrusion-based 3D printer, DLP was utilized.^12^ In the study of Levato et al.,^43^ the DLP technique was used to print complex shapes of GelMA with high fidelity. Due to its higher resolution, the literature indicates that higher infiltration and migration of surrounding cells to the hydrogel could be achieved with DLP compared to the FDM technique.^12^

Anti-inflammatory agent, the IL-4-loaded radially oriented GelMA scaffold with a pore size of 100–200 μm, was printed via DLP printing for chondrogenic differentiation. To mimic the osteochondral layer, the HAp/PCL scaffold was printed at 70 °C by conventional FDM printing to stimulate osteogenic differentiation. GelMA and HAp/PCL layers were successfully bound together by exposure to UV light (30 mW/cm^2^) for 10 s. IL-4 reduced the negative effects of inflammation on murine chondrocytes which were previously observed in other studies as well.^63−65^*In vitro* studies indicated that the expression of the chondrogenic genes aggrecan and Col2-α1 was significantly decreased in the IL-1 ß-treated group after 2 days. However, the addition of IL-4 reduced the effect of IL-1ß on chondrocytes, which considerably increased the expression of aggrecan and Col2-α1.^12^

Furthermore, reinforcing GelMA hydrogels with melt-electrowritten polyester fibers was an approach to improving mechanical properties. One drawback of PLA/PLGA materials compared to PCL was their high melting temperatures compared to PCL (PLA: ∼180° and PLGA: ∼130°), which can make the co-printing of live cells with PLGA/PLA a challenge.^66^ Therefore, to fabricate durable implants and reinforce mesenchymal stem cell (MSC)-laden hydrogels, 3D printing was first used to create networks of PCL, PLA and PLGA (85:15 and 65:35) fibers.^52^

Articular cartilage has collagen fibers with a diameter in the range 50–300 nm. Electrowritten fibers mimic closely the aligned collagen fibers in the cartilage zone of subchondral tissue. Hydrogels may, therefore, be reinforced with microfibers with electrowriting technology.^3,67^ When PCL microfibers were deposited on relatively more conductive GelMA hydrogels with melt electrowriting, larger diameter fibers (11 μm diameter) were achieved compared to ones deposited on nonconductive surfaces such as PCL.^3^ Qiao et al.^67^ reinforced GelMA hydrogels with a triblock polymer of PCL and PEG (PCEC) microfiber (fiber diameter 15 μm).^67^ These microfibers are much larger than the collagen fibers in the articular cartilage. Then, PLGA microspheres were loaded with BMSCs and then incorporated in the microfiber reinforced GelMA hydrogels. Afterward, a triphasic construct was fabricated by addition of bone morphogenetic protein-7 (BMP-7)/TGF-β1, TGF-β1 and BMP-2 to cartilage, calcified cartilage and subchondral bone zones, respectively. The study indicated that BMSCs were capable of differentiating both to chondrocytes and to osteoblasts in the relevant zones.^67^ PCR study indicated that Col2-α1, Sox-9 and superficial zone protein were expressed for the cartilage zone. The calcified cartilage zone expressed Col2-α1 and aggrecan, and the bone layer expressed alkaline phosphatase (ALP) and osteocalcin, which indicated successful biomimicry of the scaffolds.

### 2.2 Alginate-Based Hydrogels

Alginate hydrogels are biocompatible, and they can hold a high amount of water, which makes them a suitable candidate for soft tissue engineering applications.^68^ Sodium alginate scaffolds have gel-like characteristics; therefore, they can keep the form of chondrocytes within their structure. Sodium alginate may be gelled with Ca ions rapidly; however, it undergoes shrinkage during this process, and its stiffness remains inadequate for osteochondral tissue.^69,70^

In some studies, alginate is blended with other polymers to enhance its mechanical properties and improve the biological response.^1,16,71^ In a number of studies, gellan gum was blended with alginate to improve stiffness.^39,69^ However, there is the possibility of nozzle clogging, and addition of a thixotropic inorganic bioactive component may improve the hydrogel printability. In the study of Chen et al.,^39^ thixotropic magnesium phosphate-based gel was incorporated in a 2.5% alginate/3% gellan gum-based bioink in a ratio of 1.5:1. 3D printing was performed with an extrusion-based printer, and scaffolds were cross-linked with 0.1 M CaCl_2_ for 15 min. The pore size of the hydrogel was in the range 10–80 μm. Incorporation of gellan gum improved the compressive modulus from 350 to 550 kPa. Thixotropic magnesium phosphate-based gel improved the shear thinning properties and injectability of the alginate/gellan gum-based bioink. High MG-63 cell viability (80%) was achieved with these scaffolds.^39^

HAp is difficult to disperse in alginate, and it may lead to clogging of the nozzle. In another study, to improve dispersion of HAp and prevent clogging of the nozzle of the 3D printer, sodium citrate was added as a dispersant to chondrocyte loaded 1–2% HAp/alginate hydrogel, which was then printed with Bioplotter (EnvisionTec). In the presence of HAp, chondrocytes secreted a calcified matrix. To preserve the hydrogel’s form, PCL was 3D printed via an extrusion-based printer, and then the hydrogels were loaded onto the PCL matrix. *In vitro* and *in vivo* studies with mice indicated that these structures were suitable for mimicking calcified cartilage.^68^

Inducing microporosity to 3D printed alginate hydrogels was also the focus of a few studies.^72,73^ Alginate has a slow degradation rate in the body, and it has a porosity in the nanometer scale. To increase the microporosity of the 2% alginate, methyl cellulose (MC) was added to serve as a porogen into the alginate scaffolds with a ratio of 1:1 and 1:2. Then the scaffolds were printed with a multi-head 3D plotter with a pressure of 0.1–0.2 MPa. These scaffolds promoted chondrogenesis of BMSCs *in vitro* and *in vivo* with mice.^72^ In another study, to mimic the articular cartilage zone, 9 w/w% MC was added in 3 wt % alginate bioink to increase the viscosity and printability of the bioinks that were then bioplotted. This formulation was found to be suitable for entrapment of human mesenchymal stem cells (hMSCs). Then, the osteochondral layer was 3D printed of calcium phosphate cement (CPC). CPC was set at 37 °C for 30 min, and alginate was cross-linked with calcium chloride solution. CPC and hydrogel were observed to have strong adhesion.^73^

Idazsek et al.^1^ 3D printed 4% w/v alginate with 6% w/v GelMA, 4% w/v chondroitin sulfate with vinyl moieties (CS-AEMA) to mimic the hyaline zone using an extrusion-based printer with a speed of 235 mm/min. Vinyl moieties were introduced via the 1-Ethyl-3-(3-dimethylaminopropyl)carbodiimide (EDC)/N-hydroxysuccinimide (NHS) coupling reaction with 2-aminoethyl methacrylate. hMSCs and human articular chondrocytes (hACs) were loaded into the hydrogels. To mimic the osteochondral layer, 4% w/v alginate was blended with 6% w/v GelMA, 4% w/v CS-AEMA, 0.5% w/v HAMA and 0.5% w/v TCP microparticles to produce the hydrogels with encapsulated hMSCs. *In vitro* studies indicated a high % cell viability of the cells for hydrogels of all bioink formulations. RT-qPCR studies indicated higher expression of Col2-α1 and aggrecan for hMSC and hAC loaded scaffolds, which indicated improvement of chondrogenic potential of hMSCs after hAC loading. Incorporation of tricalcium phosphate (TCP) microparticles increased the expression of Col1 α1 and ALP expressions for the osteochondral layer.

Gelatin is added in alginate hydrogels to improve their mechanical integrity.^16,71^ In the study of Yang et al.,^16^ 5% gelatin/8% alginate and 5% gelatin/8% alginate/4% HAp (with a pore size of 200 μm) as cartilage and bone layer, respectively, were 3D bioprinted with an extrusion-based printer at 37 °C to produce bilayer scaffolds. Before cross-linking alginate, addition of 5% gelatin was found to help keep the integrity of the scaffold structure which otherwise collapsed. Alginate was then cross-linked with calcium ions to form a stable gel. *In vivo* studies with a rabbit model indicated the repair of full thickness articular cartilage. In the study of Joshi et al.,^71^ 2.5 w/v % silk was incorporated in 2.5 w/v % alginate and 5 w/v % gelatin and printed with a multi-nozzle extrusion-based printer at 21 °C with a pressure of 110 kPa. The addition of silk improved chondrogenesis of hMSCs. Additionally, this composition had an adequate viscosity for printing; however, further addition of gelatin than 5 w/v % gelatin led to increase of viscosity which reduced printability.

One of the important focuses of designing osteochondral tissue scaffolds is achieving a strong bond between the layers as well as between the scaffold and the bone.^6,29,74^ In addition to designing multilayered components, in some studies fibrin glue is utilized to bond layers.^29^ In one study, GelMA/alginate hydrogels were 3D printed with a FDM printer to serve as the cartilage layer. The calcified cartilage layer was composed of 0.5% β-TCP/GelMA/alginate, and osteochondral bone was composed of PCL. Interestingly, the layers were held strongly with fibrin glue.^29^ Gradient structures also enable strong adhesion between layers. To mimic the gradient structure of osteochondral tissue, Zhang et al.^6^ 3D printed a double hydrogel network of alginate/acrylamide hydrogel with varied nano-sized HAp concentrations (0, 40, and 70 w/w% for cartilage, calcified cartilage, and subchondral bone mimics, respectively) and cross-linked the gel with calcium chloride. The scaffold had excellent mechanical properties due to strong network entanglement.

In the study of Zhu et al.,^74^ rather than using HAp, mesoporous bioactive glass (MBG) was used to reinforce the alginate hydrogel to 3D print (EnvisionTec) a triphasic structure. The cartilage layer was composed of alginate cross-linked by soaking in calcium chloride solution, the middle layer was a dense MBG/alginate, and the bone layer was a porous alginate/MBG scaffold. The results of the study indicated that a high interfacial bonding strength was achieved between layers.

**Figure 6:**
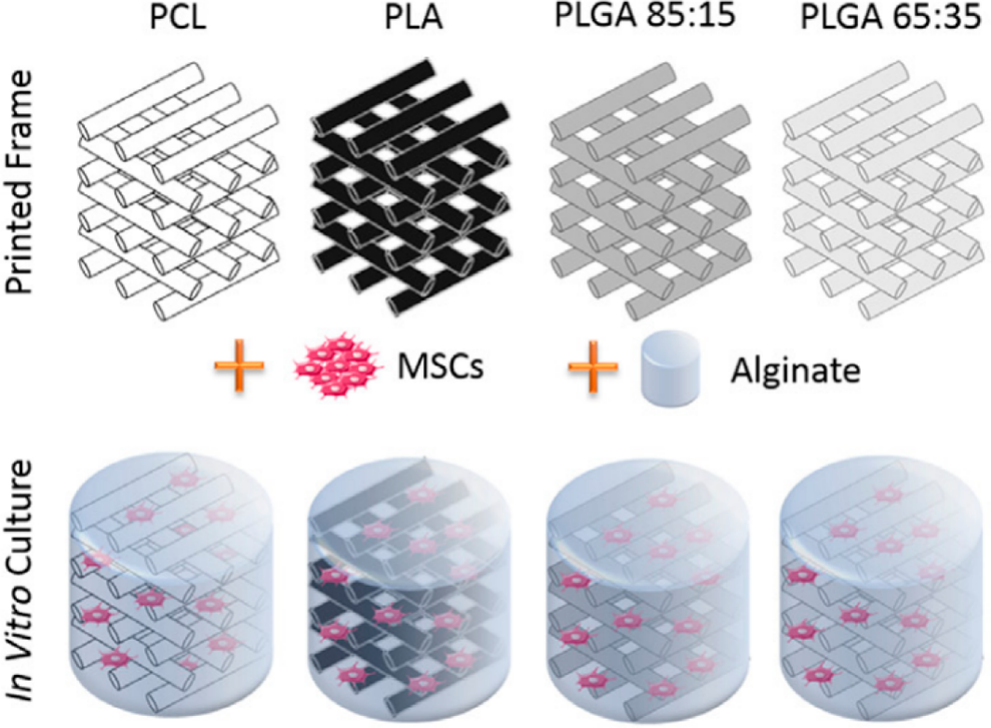
Polyester fiber reinforced MSC laden-alginate constructs produced with 3D printing^52^ (Reproduced with permission from ref (52). Copyright 2020 Elsevier).

In the study of Critchley et al.,^52^ biphasic constructs were 3D printed (RegenHU) where the bone layer was composed of agarose or γ-irradiated alginate hydrogel loaded with MSCs and the cartilage layer was composed of chondrocytes and fat pad-derived stem cells entrapped in γ-irradiated alginate hydrogels. The hydrogels were reinforced with 3D printed polyester fiber networks including PCL, PLA, and PLGA (85:15 and 65:35). PCL was chosen over PLA and PLGA due to its lower printing temperature. Figure 6 shows the constructs that reinforce alginate hydrogels with polyester scaffolds.

Calcium Col1 was present in the MSC-laden osteochondral regions and in the alginate/PCL group without cells. *In vivo* studies were conducted with the scaffolds prepared. The bone region had vascularization, and the cartilage was observed to be avascular with a hyaline cartilage-like appearance.^52^

### 2.3 Other Hydrogels

Although GelMA and alginate hydrogels were more commonly used for osteochondral tissue regeneration, other hydrogels are also successfully used including PEGDA, cellulose-based hydrogels, HAMA and PU which will be discussed in this section.

PEGDA is a photo-cross-linkable polymer.^18,75^ In the study of Hao et al.,^76^ 3D printed, micropatterned 20–30% PEGDA microscaffolds containing 1% MC were produced with a customized DLP-based SLA and the chondrocytes were laden in a collagen hydrogel. 0.05% lithium phenyl 2,4,6-trimethylbenzoyl phosphinate (LAP) was used as the photoinitiator, 0.05% tartrazine was used as a light absorber, and 0.05–0.2% nanocellulose fiber was incorporated in the hydrogels. Hydrogels with different structures (triangle, square and hexagonal) were produced and hexagonal-shaped microscaffolds. Cell culture studies with BMSCs showed that the hydrogels were cytocompatible and indicated that hexagonal-shaped scaffolds had the best cell coverage rate (73.3%).^76^

In the study of Zhou et al.,^18^ GelMA and PEGDA were utilized in the preparation of a primary ink (GelMA-PEGDA) with a tabletop SLA printer. PEGDA was blended with GelMA to improve the strength and printability of the scaffolds. Irgacure 2959 (I2959) photoinitiator was used for stabilizing and strengthening the hydrogels. The cartilage layer was composed of TGF-β1 loaded PLGA nanoparticles (120 nm diameter) for controlled release, and these nanoparticles were incorporated in GelMA-PEGDA hydrogel. The bone layer was comprised of HAp incorporated in the GELMA/PEGDA hydrogel. hMSCs were seeded on the scaffolds due to their inducibility properties for osteochondral tissue repair.^18^ The expression of chondrogenesis associated genes (Sox-9, Col2-α1 and aggrecan) increased significantly with the addition of TGF-β1. There was no chondrogenesis in the scaffold without TGF-β1. Therefore, the authors suggested that TGF-β1 has a vital role in the chondrogenic differentiation of hMSCs.

**Figure 7:**
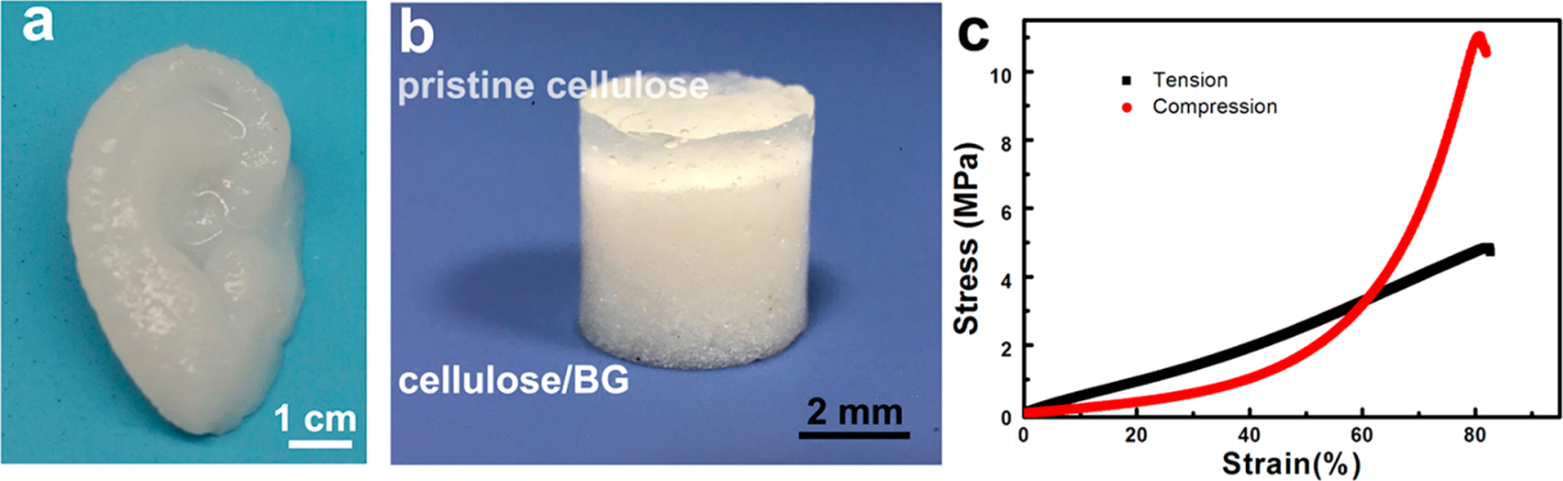
Schematic illustration of biomaterials: (a) 3D printed ear-like construct with BG incorporated cellulose ink; (b) bilayer of pristine cellulose and BG/cellulose hydrogel; (c) stress–strain curve of the 3D printed scaffolds under tension and compression^77^ (Reproduced with permission from ref (77). Copyright 2022 ACS Publications).

Cellulose-based hydrogels also were successful in osteochondral repair. In the study of Guo et al.,^77^ ear-like constructs and bilayer cellulose scaffolds were prepared by an extrusion-based 3D bioplotter as shown in Figure 7(a) and (b), respectively. Figure 7(c) shows the stress–strain curves under both tension and compression. During the mechanical loading process, the hydrogen bonds of the cellulose network dispersed the energy, and epichlorohydrin cross-linked covalent bonds sustain the integrity of the cellulose hydrogel which avoids stress concentration. The bone layer was mimicked by adding bioactive glass (BG) into the cellulose ink which has strong osteoconductivity. *In vivo* studies with NZW rabbit indicated that the shear bonding strength of bilayered cellulose scaffolds was found to be 0.56 and 0.91 MPa after 4 and 8 weeks, respectively. A strong osteointegration increased the bonding interaction between the bone and the bilayer scaffold, while the pure cellulose gel could not. The bone was found to be grown 200 μm deep into the BG/cellulose layer.

**Figure 8:**
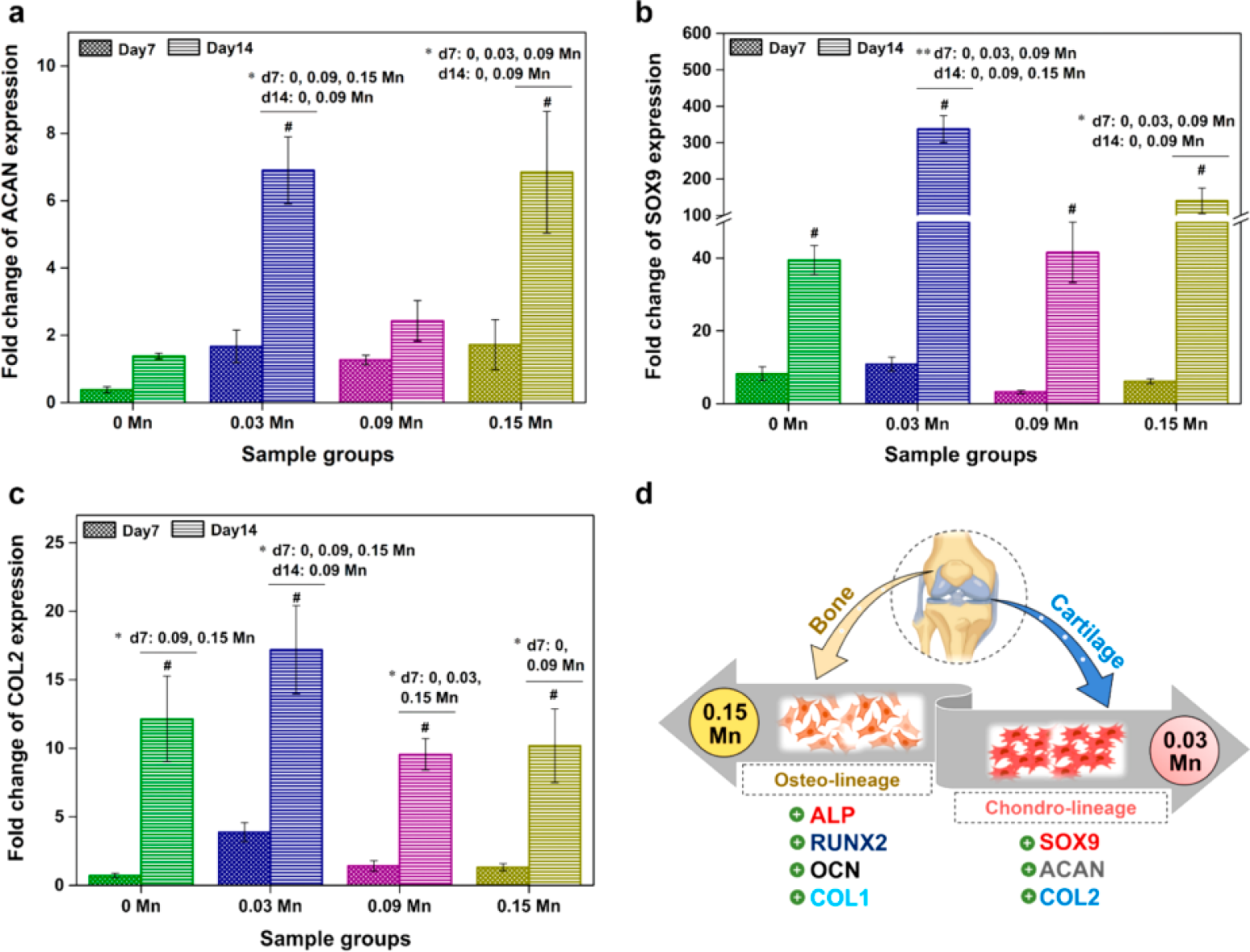
Gene expressions of cartilage specific markers for different dopings of Mn ion in CaP in CaP/HPMC ceramic ink: (a) ACAN (aggrecan); (b) SOX9; (c) Col2-α1; (d) expressed genes and specific lineage of ceramic inks with 0.15 and 0.03 Mn^10^ (Reproduced with permission from ref (10). Copyright 2022 ACS Publications).

Bilayered or multilayered scaffolds are found to be usually inefficient in biomimicking the osteochondral tissue, as delamination may occur between the layers.^10^ Kamaraj et al.^10^ prepared a ceramic ink by mixing sintered calcium deficient apatite ceramic powders with hydroxypropyl methylcellulose (HPMC). Calcium deficient apatite had different molar concentrations of Mn ions including 0.03, 0.09, and 0.15 mol/L, which were denoted as 0.03 Mn, 0.09 Mn and 0.15 Mn, respectively. HPMC functioned as a polymeric binder, and the bioink was 3D printed with an extrusion-based printer (BioBots) with an interconnected microporous structure. Without the use of any chemicals or growth factors, Mn doping in the CaP ceramic increased cell proliferation and differentiation of hMSCs into the osteogenic and chondrogenic phenotypes. Figure 8 shows the gene expression for different loadings of Mn in the CaP/HPMC scaffolds.

Mn was found to enhance cell adhesion by opening the TGF-ß binding site. It is evident that 0.15 mol/L Mn ion doping leads to a higher level of expression of bone-specific markers. For chondrogenesis, Sox-9 genes had higher expression for 0.03 mol/L Mn ion doped CaP/HPMC samples.^10^

HAMA^62,78,79^ chondroitin sulfate-based hydrogel,^80−82^ and PU^83,84^ hydrogels were also studied to mimic the cartilage layer of osteochondral tissue scaffolds. Since HAMA contains GAG, it holds high amounts of water and dissipates the energy during loading, which makes it suitable for osteochondral regeneration.^56^ Since its mechanical properties are poor, it is usually used as an additive with GelMA or alginate hydrogels to improve chondrogenic potential, as mentioned in the previous section in detail.^1,54^ Chondroitin sulfate also has poor mechanical properties, and it is used together with GelMA and PEGDA hydrogels to reduce immune response and improve cell viability and chondrogenic differentiation.^80−82^

PU is biocompatible and biodegradable and has high elasticity and tensile strength, which makes it suitable for cartilage regeneration.^85−87^ Wen et al.^84^ prepared bioinks of PU and added poly(ethylene oxide) to improve their printability. PU microspheres with stromal cell-derived factor-1 (SDF-1) and Y27632 (a small molecular drug) loading were also incorporated in the bioinks. SFD-1 and Y27632 both trigger chondrogenic differentiation of MSCs.^84^ SDF-1 triggers MSCs homing and migration.^89,90^ Therefore, the literature suggests that using SDF-1 may eliminate the need of loading hydrogels with MSCs which has a risk of contamination.^88^ The bioinks were 3D bioprinted (Regenova) with a weight ratio of 54:22:24 for PU:PU microspheres:poly(ethylene oxide) at −40 °C under 230 kPa. qRT-PCR studies indicated that the expression of Sox-9, aggrecan and Col2-α1 significantly increased for SDF-1 and Y27632 loaded PU microsphere incorporated scaffold groups compared to PU.

Chitosan-based hydrogels are another alternative; however, chitosan requires low pH levels for its dissolution, which are toxic for cells.^91,92^ Therefore, there is less research with chitosan-based hydrogels for osteochondral regeneration compared to other natural hydrogels.^34^ Moreover, elastin-based^23,93^ and fibrin-based hydrogels^94^ were also studied for articular cartilage repair. However, elastin-based hydrogels easily calcify, which makes them less suitable for osteochondral repair. On the other hand, fibrin is highly expensive, which makes it unfavorable despite its high performance.

## 3 3D Printed Polyester Scaffolds

The stability of the hydrogels may not be sufficient to support osteochondral tissue regeneration. To solve this problem, in many studies, rather than using hydrogels with polyester fiber reinforcement, solely polyester fiber scaffolds were prepared for osteochondral tissue regeneration.^95^ In this section, the research on polyesters without use of hydrogels is discussed. PCL is one of the most commonly studied polyester in the field of osteochondral research^96,97^ In most of the studies, PCL is usually produced with an extrusion-based bioprinter. PCL is preferred due to its low processing temperature, biocompatibility, and mechanical stability.^98^ In the study by Basal et al.,^35^ graphene incorporated 3D printed PCL scaffolds were produced. A needle with a 410 μm diameter was used and printing was carried out with a printing speed of 12 mm/s and 3 bar air pressure. Graphene was introduced into the scaffold due to its inductive effect on both bone and cartilage tissue repair. *In vivo* studies with NZW rabbit were conducted, and histology results indicated that the 10 w/w% graphene/PCL group had the most successful healing among the study groups. The 10 w/w% graphene/PCL group had the highest expression of vascular endothelial growth factor (VEGF), ALP, BMP-2 and Col-1.

Li et al.^7^ used a novel printing method to produce PCL/polyvinylpyrrolidone (PVP) scaffolds. To increase the resolution of printing, an electrohydrodynamic jet printer was used, and this technique led to the production of much finer fiber size and improved resolution of printing. A higher resolution of printing is important to be able to precisely mimic cartilage tissue. Li et al. combined PCL with PVP and optimized the flow rate, applied voltage, printing height, and viscosity of the biopolymeric ink, as these have a major effect on scaffold morphology. The samples were printed at 55 °C, and the results indicated that electrodynamic jet printed scaffolds had strong interlaminar bonding.

Decellularized extracellular matrix is also commonly 3D printed together with polyesters, since it triggers chondrogenesis.^26,98,99^ In the study by Gruber et al.,^98^ PCL and PLA were printed by traditional mechanical FDM. With mechanical FDM, the substrate was extruded via drive wheels rather than pneumatic pressure. It would be advantageous to prepare two layers at once by a single-fit implant which encourages the formation of cartilage and underlying bone together. *In vitro* studies indicate that decellularized extracellular matrix is chondro-inductive as well.^100−102^ To improve the chondrogenesis, decellularized matrices were incorporated in PLA microspheres which were then mixed with PCL and 3D printed via an FDM printer at 65–70 °C.^26^

In a number of studies, PCL was reinforced with HAp^103−105^ or bioglass^106^ in bilayered scaffolds for constructing the bone layer, which led to osteogenic differentiation and enhancement of stiffness. PLA was also reinforced with europium functionalized 10% nano-sized HAp for construction of the bone layer. The scaffolds were found to improve chondrogenesis and osteogenesis of progenitor cells isolated from adipose tissue.^107^

Camacho et al.^56^ 3D printed peptide (amino acid sequence: RYPISRPRKR) and mineralizing peptide E3 conjugated PCL with an extrusion-based printer with a nozzle diameter of 100 μm and a pressure of 70 psi. Peptide with the RYPISRPRKR amino acid sequence is from the hyaluronic acid binding sequence of aggrecan and induces MSCs’ chondrogenesis, and mineralizing peptide stimulates hMSCs’ osteogenic differentiation. In this study, additionally, the two peptides were conjugated at once to PCL, which was shown to promote the differentiation of MSCs to articular and hypertrophic chondrocytes. Table 1 shows an overview of the constructed scaffolds for osteochondral regeneration.

**Table 1:** Material Choice with Relevant 3D Printing Technique for Each Layer of Osteochondral Tissue and Their *In Vitro* and *In Vivo* Studies

**Chondrogenic layer (material/printing technique)**	**Calcified cartilage layer (material/printing technique)**	**Osteochondral layer (material/printing technique)**	***In vitro/in vivo*****studies**	**Ref**
GelMA, PEO (bMSC) /DLP				(43)
PCEC fibers, GelMA (BMP-7, TGF-β1, BMSC) /MEW	PCEC fibers, GelMA (TGF-β1, BMSC loaded)/MEW	PCEC fibers, GelMA, BMP-2 (BMSC loaded)/ MEW	*In vivo*: NZW rabbits	(67)
TMP in alginate, gellan gum (MG-63 loaded)/extrusion			*In vivo:* NZW rabbits	(39)
	HAp, alginate, PCL (chondrocyte loaded)/extrusion		*In vitro*: chondrocyte, *in vivo:* mice	(68)
15% GelMA/extrusion	20% GelMA, nano-sized HAp/extrusion	30% GelMA/nano-sized HAp/extrusion	*In vitro:* BMSCs, *in vivo:* NZW rabbits	(2)
GelMA (TGF-β1/extrusion		HAp in GelMA/extrusion	*In vitro*: BMSC, i*n vivo:* rats	(57)
20% PRP, GelMA/SLA		2% PRP, GelMA/SLA	*In vitro*: BMSC	(27)
Radially oriented GelMA (IL-4 and L929) /DLP		HAp, PCL/extrusion	*In vivo:* NZW rabbits	(12)
GelMA, xanthan gum/SLA		β-TCP in GelMA with xanthan gum/SLA	*In vitro:* hMSCs	(108)
MC with alginate (chondrocyte)/3D bioplotted		CPC	*-*	(73)
Alginate/CS (hMSC and hACs)/extrusion		TCP, GelMA, CS-AEMA, HAMA (hMSC and hACs)/extrusion	*In vivo*: rats	(1)
Gelatin, alginate/extrusion		HAp, gelatin, alginate (BMSC)/extrusion	*In vitro*: BMSCs, *in vivo:* NZW rabbits	(16)
Alginate, acrylamide (BMSC loaded)/extrusion	Alginate, acrylamide, 40% nano-sized HAp (BMSC loaded)/extrusion	Alginate, acrylamide, 70% nano-sized HAp (BMSC loaded)/extrusion	*In vivo:* rat model	(6)
Alginate, silk, gelatin (hMSC loaded /multi-nozzle extrusion		Alginate/silk/gelatin (MG-63 loaded)/multi-nozzle extrusion	*In vitro*: hMSC	(71)
dCECM, GelMA, Alginate hydrogel (adipose-MSC)/extrusion			*In vivo:* NZW rabbits	(109)
NAGA, GelMA/extrusion			*In vitro:* BMSC, *in vivo*: N/A	(110)
Sodium alginate/extrusion	SA, MBG/extrusion	Porous SA, MBG/extrusion		(74)
Alginate, PLA fiber/coaxial extruder	Alginate, GelMA, β-TCP/coaxial extruder	PCL/coaxial extruder		(29)
γ-alginate with PCL, PLA or PLGA struts (BMSC)/extrusion		Agarose or γ-alginate hydrogel with PCL, PLA or PLGA struts (BMSC)/extrusion	*In vivo*: nude mice	(52)
MC, PEGDA (chondrocyte)/DLP				(76)
GelMA, PEGDA (GO-TGF-β1 in PLGA microspheres, hMSC loaded) /SLA		HAp in GO loaded GelMA, PEGDA hydrogel (hMSC laden)/SLA	*In vitro:* MSCs	(111)
Cellulose hydrogel/extrusion		BG in cellulose hydrogel/extrusion	*In vivo:* NZW rabbits	(77)
HPMC, 0.03% Mn doped CaP powder/extrusion		HPMC, 0.15% Mn doped CaP powder/extrusion		(10)
		10% graphene, PCL/extrusion	*In vivo*: NZW rabbits	(35)
		PCL, PVP, PEG/electrodynamic jet printing		(7)
Decellularized matrix, PCL/bioplotted		TCP, PCL/bioplotted	*In vitro* hMSC	(98)
Chitosan, PCL, PLGA/extrusion		β-TCP, PCL, PLGA/extrusion	*In vitro*: MSCs	(46)
HNP or HMP, akermanite/extrusion		HNP or HMP, akermanite/extrusion	*In vitro*: rBMSCs, in vivo: NZW rabbits	(112)

In the native osteochondral tissue, the compressive modulus values of cartilage and subchondral bone are 2–20 MPa and 98–270 MPa, respectively.^29,113^Table 2 shows that by incorporation of polyester fibers for the reinforcement of hydrogels, higher mechanical properties could be achieved.

**Table 2:** Compressive Moduli of Articular Cartilage and Scaffolds Prepared with 3D Printing Techniques for Cartilage Regeneration

	**Materials choice**	**Pore size (μm)**	**Compressive modulus**	**Ref**
	Native human articular cartilage	N/A	∼1 MPa	(114−116)
extrusion	PLA/PCL+CS/SF	N/A	1.07 GPa	(117)
	PLA template and cross-linked ALG hydrogel	110–200	228 MPa	(118)
	PLCL/poly(l-lactic acid) (PLLA)	250–500	97 MPa	(119)
	PCL/PLGA/CS	600–1800	30.2 MPa	(46)
	CS/Gel/hyaluronic acid/graphene	20	7.5 MPa	(22)
	PCL struts with GelMA hydrogel	N/A	7.24 ± 0.79 MPa	(115)
	N-acryloyl glycinamide (NAGA)/GelMA	N/A	5.5 MPa	(110)
DLP	PEGDA	N/A	4 MPa	(120)
extrusion	Hyaluronic acid/PLA	600	3 MPa	(121)
	PU scaffolds with PU microspheres	3–5	1.20 ± 0.03 MPa	(84)
	Alginate/gellan gum with TMP (1.5:1)	15–80	450 kPa	(39)
	Phosphate grafted ALG, silk fibroin solution and gel	N/A	390 kPa	(71)
	PCEC reinforced GelMA with PLGA microspheres	N/A	283.6 ± 22.3 kPa	(67)
SLA	PRP-GelMA hydrogel	127	184 KPa	(27)
extrusion	GelMA	100–400	130 kPa	(122)
	Alginate reinforced by short PLA fibers	N/A	33.39 kPa	(29)

**Figure 9:**
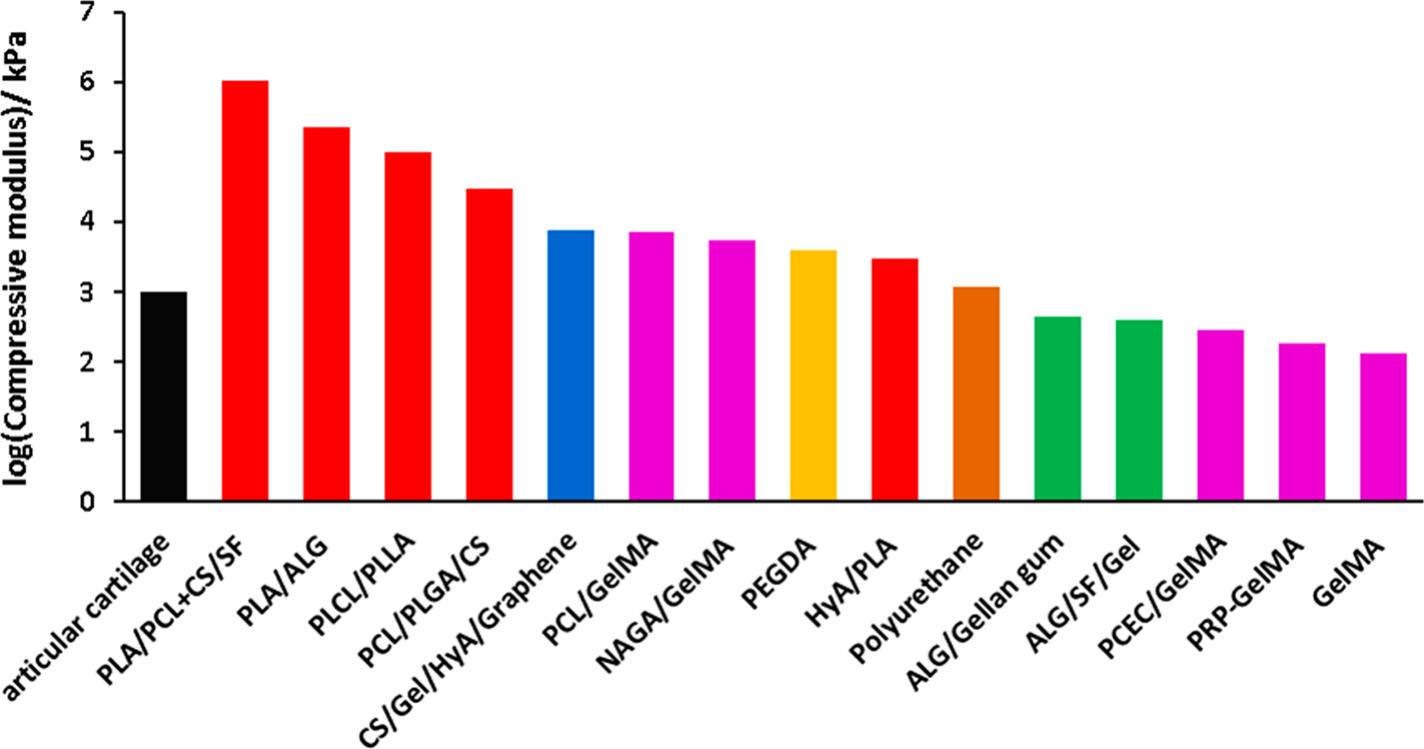
log(compressive modulus values) versus the biomaterials selection for articular cartilage design (black, articular cartilage; red, polyesters; blue, chitosan; pink, GelMA; yellow, PEGDA; brown, polyurethane; green, alginate) (drawn in Excel).

As can also be observed from Table 2, reinforcing the hydrogels with a polyester fiber or matrix is very effective in attaining the required mechanical properties. These results are also illustrated in Figure 9.

Due to the large variation of compressive modulus values, a compressive modulus graph was drawn in the log scale. Figure 9 clearly shows that biomaterials with polyesters have higher compressive modulus values than those of hydrogels. As long as GelMA or alginate had reinforcement, It was observed to have sufficient compressive modulus for articular cartilage regeneration.

### 3.1 Limitations and Future Perspectives

The literature indicates that there is a high variation of the porosity of the produced scaffolds, which is also observed from Table 2. However, the porosity of the scaffolds is critical and has a major role on osteochondral tissue regeneration. Therefore, there should be further focus on the porosity of the scaffolds, and it needs to be standardized in the future.

Moreover, electrohydrodynamic jet printing comes forth as an advanced technique to obtain higher resolution; however, there is only a limited amount of research with this technique. In the future, further research efforts with electrohydrodynamic jet printing may enable the production of scaffolds with enhanced properties for osteochondral tissue.

For multilayer scaffolds, mismatch strain is a huge challenge which may lead to failing of the design. The mechanical properties should be compatible between layers when designing 3D printed scaffolds to obtain higher adhesive strength.^33^ Moreover, adhesive strength is less frequently studied than compressive strength and it needs to be further investigated. Gradient structures are observed to be effective to mimic the osteochondral tissue; therefore, it may be more promising to focus on these structures rather than multilayer scaffolds to achieve stronger adhesion of layers.

In studies, MSCs are usually loaded in the hydrogels for promoting osteochondral regeneration; however, loading two cell types together such as MSCs with hACs may enhance osteochondral regeneration.^1^ Additionally, rather than cells, polydopamine^55,123−125^ and cytokines such as SDF-1 may be loaded in hydrogels, which also trigger chondrogenic differentiation and reduce the risk of contamination.^84^ However, more research is required on this topic. So far, *in vivo* studies have been conducted with rats or rabbits; however, *in vivo* studies with larger animals such as sheep are required to further evaluate the efficiency of these constructs for osteochondral repair.

## 4 Conclusion

In the literature, many studies on mimicking osteochondral tissue have been published. This review indicates that PCL, cell laden GelMA and alginate hydrogels were most frequently studied to mimic the cartilage layer. To enhance chondrogenesis, growth factors were also heavily used, such as TGF-β1 and SDF-1. Blending GelMA or alginate hydrogels with decellularized matrix, silk, hyaluronic acid, and chondroitin sulfate also enhanced chondrogenesis. To mimic the osteochondral layer, CaPs and bioactive glass were frequently introduced, and osteogenesis was further enhanced by addition of growth factors such as BMP-1 and BMP-2.

Triphasic and gradient structures were found to be more successful in repairing osteochondral defects. 3D printing of gradient structures is also powerful to achieve better interlaminar bonding. To strengthen the cartilage layer, PCL, PLA or PLGA fibers were successfully used as a reinforcement. Direct electrowriting was also practiced to produce polyester microfibers to reinforce the hydrogels so they can withstand the load more successfully after implantation. By using more advanced 3D printing techniques such as DLP and electrohydrodynamic jet printing, scaffolds may be produced with higher resolution, which mimics more closely the osteochondral tissue structure. In the future, *in vivo* studies with larger animal models are required to evaluate the success of these scaffolds for osteochondral repair. Overall, this review indicates that 3D printing technology holds great promise for regenerating osteochondral tissue effectively.
